# Guest-Host Complex Formed between Ascorbic Acid and β-Cyclodextrin Immobilized on the Surface of an Electrode

**DOI:** 10.3390/molecules19055952

**Published:** 2014-05-09

**Authors:** María Teresa Ramírez-Silva, Manuel Palomar-Pardavé, Silvia Corona-Avendaño, Mario Romero-Romo, Georgina Alarcón-Angeles

**Affiliations:** 1 Departamento de Química, Universidad Autónoma Metropolitana Iztapalapa, Av. San Rafael Atlixco #186, Col. Vicentina, Mexico D.F. C.P. 09340, Mexico; E-Mail: mtrs218@xanum.uam.mx; 2 Departamento de Materiales, Universidad Autónoma Metropolitana Azcapotzalco, Av. San Pablo #180, Col. Reynosa-Tamaulipas, Mexico D.F. C.P. 02200, Mexico; E-Mails: scav@correo.azc.uam.mx (S.C.-A.); mmrr@correo.azc.uam.mx (M.R.-R.); 3 Departamento de Sistemas Biológicos, Universidad Autónoma Metropolitana Xochimilco, Calzada del Hueso 1100, Col. Villa Quietud, Delegación Coyoacán, Mexico D.F. C.P. 04960, Mexico; E-Mail: ageorgina@hotmail.com

**Keywords:** ascorbic acid, β-cyclodextrin, polymerization, surface inclusion complex

## Abstract

This work deals with the formation of supramolecular complexes between ascorbic acid (AA), the guest, and β-cyclodextrin (β-CD), the host, that was first potentiodynamically immobilized on the surface of a carbon paste electrode (CPE) throughout the formation of a β-CD-based conducting polymer (poly-β-CD). With the bare CPE and the β-CD-modified CPE, an electrochemical study was performed to understand the effect of such surface modification on the electrochemical response of the AA. From this study it was shown that on the modified-CPE, the AA was surface-immobilized through formation of an inclusion complex with β-CD, which provoked the adsorption of AA in such a way that this stage became the limiting step for the electrochemical oxidation of AA. Moreover, from the analysis of the experimental voltammetric plots recorded during AA oxidation on the CPE/poly-β-CD electrode surfaces, the Gibbs’ standard free energy of the inclusion complex formed by the oxidation product of AA and β-CD has been determined for the first time, ∆G^0^_inclus_ = −36.4 kJ/mol.

## 1. Introduction

**Figure 1:**
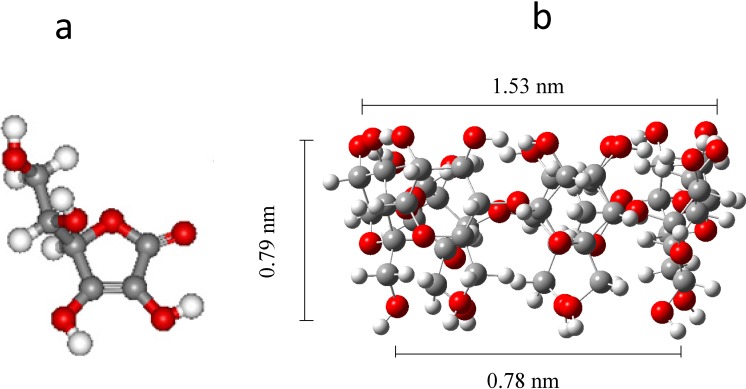
Molecular structures of: (**a**) Ascorbic acid, (5*R*)-[(1*S*)-1,2-dihydroxyethyl]-3,4-dihydroxyfuran-2(5*H*)-one, and (**b**) β-cyclodextrin, C_42_H_70_O_35_ [27].

Ascorbic acid, AA, also termed vitamin C (Figure 1a), is quite an important biocomponent widely present in living organisms [1] as anti-oxidation promoter. However, such a wide scale presence in diverse biological media can also be seen as the origin of interference problems, particularly when analytical research studies aim to effect quantitative determinations of other biologically relevant analytes, which happen to be present in much smaller concentrations, but that are nonetheless specifically important, such as the catecholamines (CAs). The latter produce, as a result of chemical or electrochemical interactions, an oxidation signal overlapping that of the AA, in particular when undertaking quantitative determinations of either and both are present in the same solution [2,3,4,5,6,7].

In chemometric terms, it is not just this last problem that needs to be contended with, but also the acute disparity of concentrations in biological samples and the actual signal separation of both analytes that has given rise to quite a significant interest in characterizing the electrochemical behavior of AA in different systems. For instance, during the quantitative determination of CAs using electrochemical techniques AA is considered an interfering analyte [8,9,10,11,12,13]. Therefore, diverse electrode surface modifying agents have been proposed in order to overcome this problem. In the studies carried out to form supramolecular complexes [14,15,16] with CAs in the presence of the AA [2,3,4,5,6,7,17], it was important to also study the supramolecular interactions that the electrode surface-modifying agents may induce in the AA, specifically when membranes are formed. Some of these studies have been done with β-cyclodextrin, β-CD, which comprises 7 α-D-glucopyranose units [18,19] (Figure 1b). Its interaction with organic, inorganic and biological molecules has seemed possible because the molecular structure of the β-CD resembles a truncated cone basket, where the inner surface of the cavity displays a hydrophobic character, and is capable of hosting some of the said molecules, provided they have the polarities and sizes adequate to fit and form guest-host-type complexes. On consideration of the aforementioned results, this work studies the influence of surface–modifying agents like β-CD in the form of its polymer poly-β-CD [20,21 on a carbon paste electrode, CPE, [22,23] used to analyze the supramolecular interactions with the AA. It is important to mention that some complex polymers that include β-CD in their chemical structure have been reported for the controlled release of drugs [24,25,26,27].

## 2. Results and Discussion

### 2.1. Electrochemical Behavior of AA over a CPE

**Figure 2:**
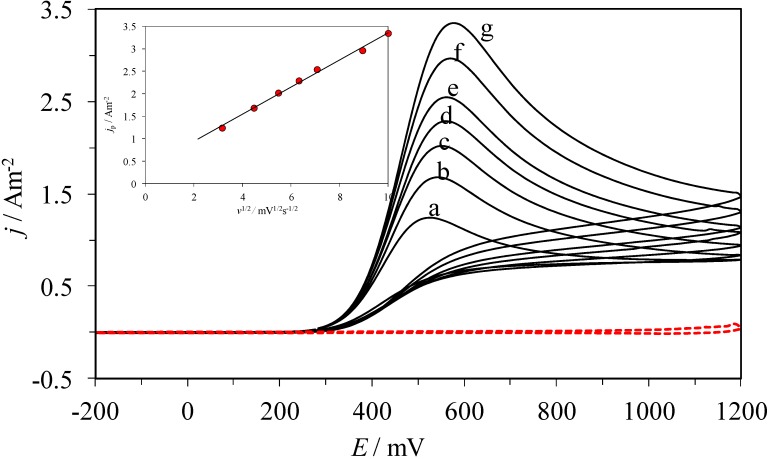
Family of cyclic voltammograms recorded in the system CPE/0.1 M NaCl, 1 mM AA at pH 3.0, at the different scan rates, ν, indicated: (a) 10, (b) 20, (c) 30, (d) 40, (e) 50, (f) 80, (g) 100, mV s^−1^. The inset shows the variation of the oxidation peak’s current density, *j*_p_, as a function of *ν*^1/2^, where the dots (˙) correspond to the experimental data and the line to the linear fitting. The broken line corresponds to the cyclic voltammogram recorded in the same system but in the absence of AA.

Figure 2 shows typical experimental cyclic voltammograms, CVs, recorded in the system CPE/0.1 M NaCl, at pH 3.0, and 1 mM AA, within the −200 to 1200 mV potential window. When the potential scan started in the anodic direction an oxidation process can be observed through the peak at *E*_pa_ = 574 mV at 100 mV s^−1^ scan rate. Subsequently, reverting the scan direction there were no reduction peaks displayed, therefore, the electrochemical process conducted is considered irreversible. When plotting the *j*_pa_ as a function of *v*^1/2^ (see the inset in Figure 2), a linear trend was obtained, thus indicating that diffusion is the rate controlling step for the oxidation process under study [28].

### 2.2. Synthesis of the β-CD Polymer, Poly-β-CD

**Figure 3:**
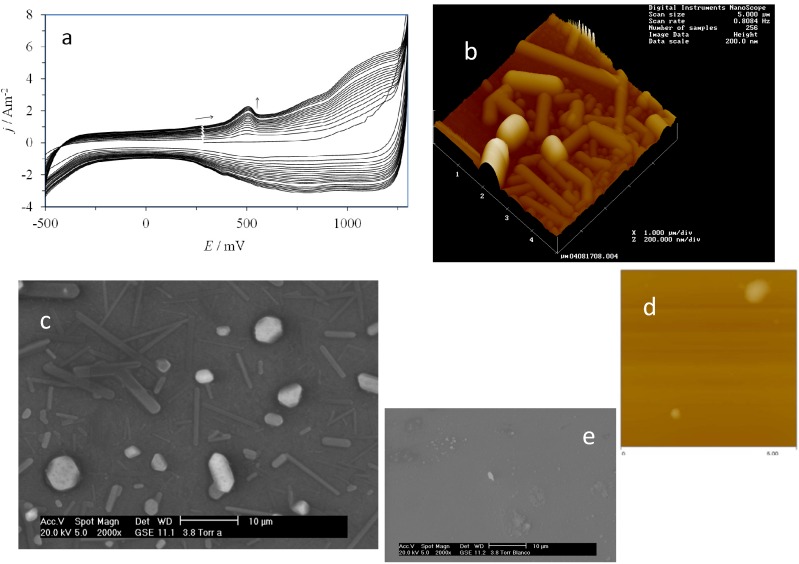
(**a**) Experimental CV plots recorded during formation of the β-CD polymer by applying 20 consecutive cycles on the surface of the CPE in a solution of 0.01 M β-CD and 1 M HClO_4_; (**b**) AFM and (**c**) SEM images taken on the surface of an indium tin oxide (ITO) electrode after the electrodeposition of poly-β-CD; The images of the bare surface of the ITO electrode are shown in (**d**) using AFM and (**e**) using SEM.

Figure 3a shows the electrochemical response recorded during the electropolymerization of β-CD over the CPE through application of 20 consecutive CV cycles. It can be clearly noted that as the cycle number increased, the current density also increased, which portrays the typical behavior of the process of electrochemical formation of a conducting polymer [29,30,31]. Moreover, as an example of the morphology of the poly-β-CD electrodeposited, Figure 3b,c show an AFM and a SEM image, respectively, of the indium tin oxide (ITO) electrode surface after the potentiodynamic deposition of poly-β-CD. It is important to note that both rod- and sphere-like poly-β-CD deposition can be recognized from the AFM and SEM images, which are related to a progressive nucleation mechanisms involving differently aged nuclei; the larger ones with spherical shapes seem to be older than the smaller rod-like deposits which grew at later stages. From concentration differences, before and after β-CD polymerization, the evaluation of the amount of β-CD molecules that were deposited was obtained. The [β-CD] was evaluated from a polarimetry calibration plot, of the specific rotation evaluated at wavelength of 545 nm, [α]_546_, as a function of [β-CD]. This way, it was found that 4.52 × 10^18^ β-CD molecules were used to form the poly-β-CD.

### 2.3. Electrochemical Behavior of AA over a CPE/Poly-β-CD Electrode

**Figure 4:**
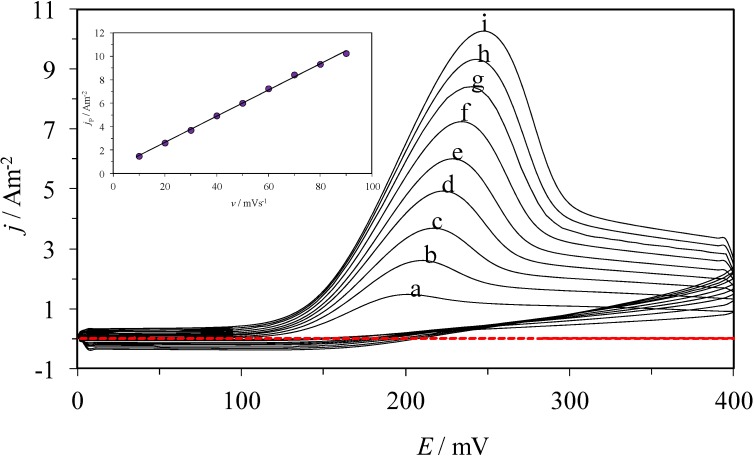
(**a**) CVs obtained from the system CPE/poly-β-CD/1 mM AA, 0.1 M NaCl, at pH 3.0, at different potential scan rates, as indicated: (a) 10, (b) 20, (c) 30, (d) 40, (e) 50, (f) 60, (g) 70, (h) 80, (i) 90, mV s^−1^. The inset shows the variation of the peak’s current density, *j*_p_, as a function of *ν*, the circles correspond to experimental data (○) of the anodic peak, while the line corresponds to the linear fitting. The broken line corresponds to the cyclic voltammogram recorded in the same system but in the absence of AA.

Figure 4 shows the family of CVs recorded in the system CPE/poly-β-CD/1 mM AA, 0.1 M NaCl at pH 3.0, at different potential scan rates. Just like in the previous case, only one oxidation peak is observed, which corroborates that the process is still irreversible, although the oxidation peak is located at about 200 mV, a comparatively smaller potential. When plotting the *j*_pa_ as a function of *v*, (see the inset in Figure 4), a linear trend is obtained in the 10 to 100 mV s^−1^ potential range, which indicates that the process is now adsorption-controlled [28].

**Scheme 1:**
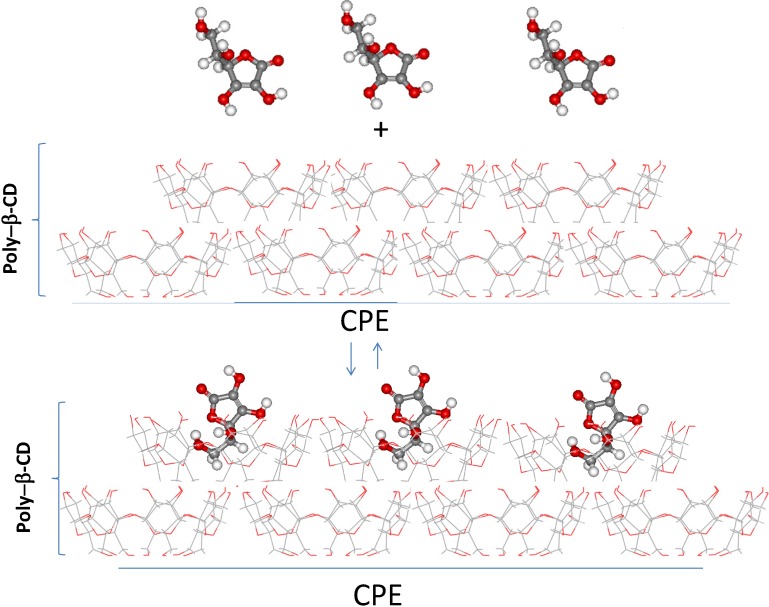
Surface inclusion complex formation between AA and β-CD immobilized on the surface of a CPE.

The AA adsorption on the CPE/poly-β-CD can be explained by the formation of an inclusion complex between the AA and the β-CD molecules that constitute the poly-β-CD polymer, see Scheme 1. Notwithstanding that Scheme 1 is a representation of the adsorption process of AA on the surfaces of the poly-β-CD modified CPE, it is based on our previous study [32] regarding a quantum chemical study of the stability of the complex formed by the neutral form of AA, that predominate at pH 3.0, where the pKa value of AA is 4.17 [33], and β-CD. In this study [32] we found that two different structures are energetically favored for this inclusion complex namely that formed by the interaction through the functional hydroxyl groups of the lactone in AA and the primary hydroxyl groups of the β-CD or the β-CD-AA complex formation through the alcohol group of AA, as is represented in Scheme 1. Furthermore, it should be added that the value of the thermodynamic constant of this inclusion complex formation has been reported by our group from both spectrophotometric and electrochemical calculations [18].

Considering that the species adsorbed are electroactive [21,32,34,35], Equation (1) has been proposed to describe the *i-E* experimental plots [34,35]:

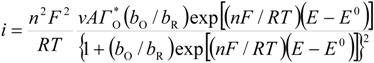
(1)where *A* is the electrode surface area, *n* is the number of electrons transferred during the heterogeneous reaction, *v* is the potential scan rate, *R*, *T* and *F* are the universal gas constant, absolute temperature and Faraday constant, respectively. *E*^0^ is the formal potential, *Γ*_о_* is the surface coverage, *b*_O_ and *b*_R_ are related with the Gibbs’ standard free energy of the surface inclusion complex formation, ∆*G*^0^_inclusion_, see Scheme 1, of the AA’s oxidized (O) and reduced (R) species, respectively.

The peaks’ potentials and the currents are given as follows:

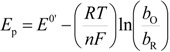
(2)

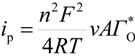
(3)


Substituting Equations (2) and (3) in (1) Equation (4) results in:

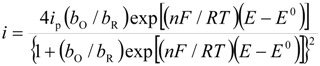
(4)

Parameterizing Equation (4) Equation (5) results in:

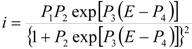
(5)
where:

*P*_1_ = 4*i*_p_ (6)

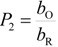
(7)

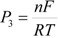
(8)

*P*_4_ = *E*^0^ (9)

**Figure 5:**
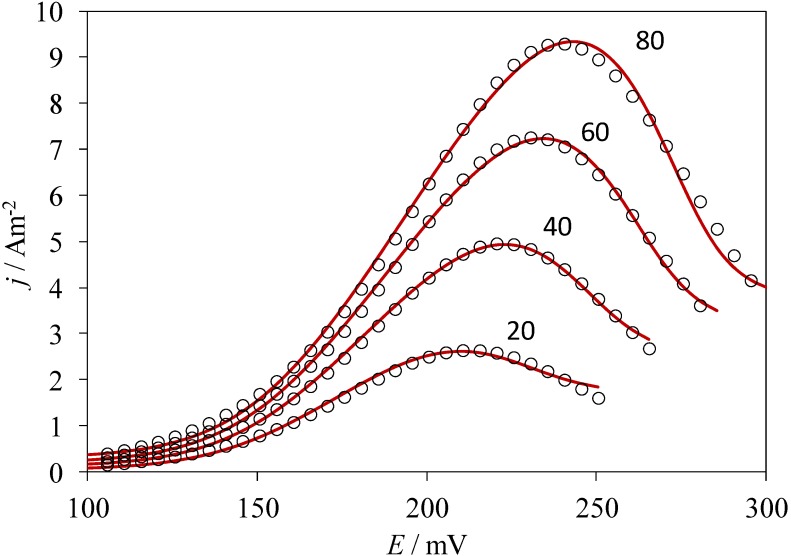
Comparison of the experimental CVs from the system CPE/poly-β-CD/1 mM AA, 0.1 M NaCl, at pH 3.0 (points) and theoretical plots (lines) generated through linear non-linear fitting of Equation (1), at different potential scan rates as indicated, in mV s^−1^.

Performing a non-linear fitting procedure to Equation (1), allows one to obtain theoretical CVs that can be compared to the experimental ones of the system CPE/poly-β-CD/1 mM AA, 0.1 M NaCl, at pH 3.0 and at 10 mV s^−1^ (Figure 5), where the adsorption model observed, derived from Equation (1), adequately describes the results from the experimental electrochemical oxidation taking place on the poly-β-CD-modified CPE. It is important to note that the fitting was quite good, regardless of the scan rate used. From this analysis, the values of the best fit parameters *P*_1_ to *P*_4_ were obtained. In particular, from the value of the parameter *P*_2_ obtained at 50 mV s^−1^ (1.7) and the thermodynamic constant value obtained through electrochemical techniques, reported in [18], for the inclusion complex between the reduced form of AA and β-CD, (ln*K*_incl._ = 8.52), the ∆G^0^_inclus._ of the oxidation product of AA was obtained as −36.4 kJ/mol. To the best of the authors’ knowledge, this is the first time that the Gibbs’ standard free energy of formation for this inclusion complex has been determined.

**Figure 6:**
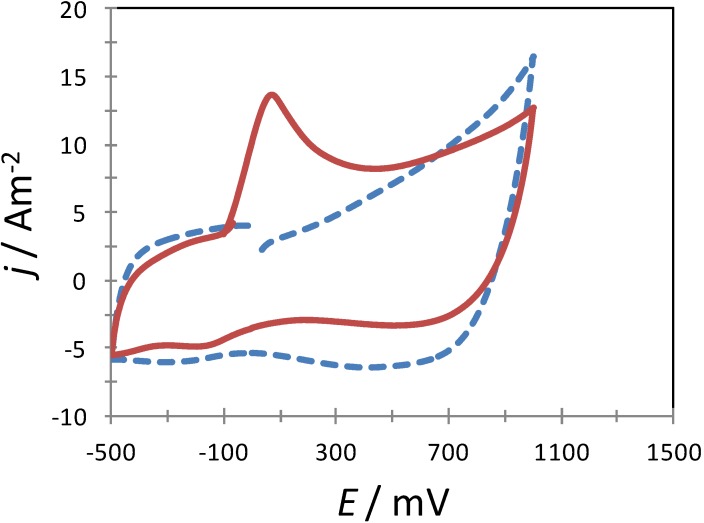
Experimental CVs recorded in the system CPE/poly-β-CD/0.1 M NaCl, at pH 3.0 (points) at 120 mV s^−1^ scan rate. In one case (solid line) the CPE/poly-β-CD was previously immersed in 1 mM AA, 0.1 M NaCl, at pH 3.0 for 5 min. whereas in the other case (broken line) the electrode was not exposed to the AA solution.

In order to corroborate further that the AA molecules were indeed adsorbed on the surfaces of the poly-β-CD-modified CPE, this electrode was immersed in an aqueous solution containing AA for a few minutes, after which the electrode was withdrawn and immediately placed in another aqueous solution containing solely NaCl to perform a CV experiment. Figure 6 depicts two experimental CVs recorded in the system CPE/poly-β-CD/0.1 M NaCl, at pH 3.0, where it is possible to note that when the CPE/poly-β-CD electrode was previously immersed in the AA solution an oxidation peak becomes apparent due to AA oxidation, otherwise there were none.

**Figure 7:**
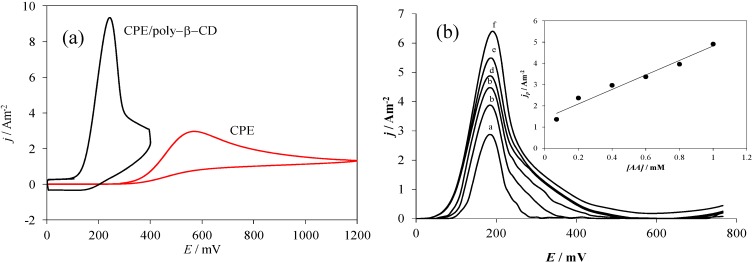
(**a**) Comparison of experimental CV plots recorded in the system electrode/0.1 mM AA, 0.1 M NaCl at pH 3.0, for different electrodes as indicated in the Figure, in both cases at 80 mV s^−1^ potential scan rate. (**b**) Family of Differential Pulse Voltammetry plots recorded in the system CPE/poly-β-CD/0.1 M NaCl, at pH 3.0 and different AA concentration: (a) 0.07, (b) 0.1. (c) 0.3, (d) 0.6, (e) 0.8 and (f) 1 mM, at a scan rate of 20 mVs^−1^, the inset show the calibration plot for AA quantification.

Figure 7a shows a comparison of CV plots recorded during AA oxidation using the electrodes considered in this work. It is possible to note that both the current density and the anodic peaks’ potential vary drastically as a function of the electrode used.

When modified electrodes were used, the respective peak potential, *E*_p,m_, moves towards less positive potential values. This change makes the difference between the peaks’ potentials of the bare carbon paste electrode, *E*_p,b_, and *E*_p,m_, ∆*E*_p_, 324 mV, see Table 1. Moreover, the ratio between the peak’s current density recorded with the modified electrodes, *j*_p,m_ and that obtained with the bare CPE, *j*_p,b_ is greater than 3. The change in the AA oxidation potential towards less positive values can be attributed to the formation of the surface inclusion complex, that apart from provoking its strong adsorption it may give rise to an increase of the heterogeneous reaction rate constant as was observed in the case of the interaction of dopamine (DA) with the same modified electrode [35]. Moreover, using a similar treatment to the one described in the present work, Palomar-Pardavé *et al.* [35], showed that the CPE/poly-β-CD/electrode can be successfully used as working electrode during the electrochemical determination of DA, in the presence of ascorbic acid at pH 3.0. Notwithstanding, in this work we shown that due to the negative value found for the ∆G^0^_inclus._, this electrode can also be adequately use for the electrochemical quantification of AA, see Figure 7b, with a sensitivity of 3.41 Am^−2^ mM^−1^ and a detection limit of 0.22 µM.

**Table 1:** Variation of the voltammetric parameters for AA oxidation as a function of the electrode used.

Electrode	*E*_p_/mV	*j*_p_/Am^−2^	*∆E*_p_ = *E*_p,b_ − *E*_p,m_/mV	*j*_p,m_/*j*_p,b_
CPE	564	2.96	0	1
CPE/poly-β-CD	240	9.19	324	3.14

## 3. Experimental

### 3.1. Reagents and Chemicals

All solutions were made from Merck (Mexico D.F., Mexico) analytical grade reagents and deionized water type 1 with 18.2 MΩcm resistivity, free from organic matter, obtained from a U.S. Filter PURE-LAB Plus UV (EU, San Diego, CA, USA). The pH was adjusted to pH 3.0 with HCl (37%). The resulting solutions were degassed with nitrogen and freshly prepared prior to each determination. They were also protected from the incidence of light even during the performance of the experiments.

### 3.2. Instrumentation

The electrochemical experiments were carried out with the aid of a potentiostat-galvanostat Autolab PGSTAT 30 (Utrecht, The Netherlands) in conjunction with a typical three-electrode electrochemical cell, with the carbon paste electrode, CPE, as working electrode. The CPE was prepared as described by Ramirez-Silva *et al.* [22,23], by mixing 1 µM, 99.9% graphite powder, from Johnson Matthey (Devens, MA, USA), with nujol. The exposed surface area of the CPE to the electrolyte solution was 0.25 cm^2^. A Pt wire was used as counterelectrode and a saturated Ag/AgCl electrode as reference, to which all potentials herein reported should be referred to unless otherwise stated. All reported measurements were recorded at 25 °C.

### 3.3. CPE Modified with Poly-β-CD

A 10 mM β-CD solution was prepared in 1 M HClO_4_ which was used to polymerize it over the CPE, as described by Roa-Morales *et al.* [20] and Corona-Avendaño *et al.* [21], by means of CV during different number of cycles. The poly-β-CD electrodeposited on an indium tin oxide (ITO) electrode was characterized *ex situ* by atomic force microscopy (AFM) applying the tapping method, with a Digital SPM Multimode instruments, Nanoscope IIIA (Santa Barbara, CA, USA) and by scanning electrode microscopy (SEM) using a JEOL JXA-8200 microscope (JEOL, Peabody, MA, USA).

## 4. Conclusions

This work presented the influence of the β-CD polymer, poly-β-CD, on the electrochemical response of AA. It was shown that the poly-β-CD drastically influenced the AA oxidation process. On the bare CPE the AA electrochemical oxidation was mass transfer-controlled while in the system CPE/poly-β-CD the process becomes adsorption-controlled, due to formation of an inclusion complex, on the electrode surface between β-CD and AA. Furthermore, the said complex catalyzed the AA oxidation reaction.

## Supplementary Materials

Supplementary materials can be accessed at: http://www.mdpi.com/1420-3049/19/5/5952/s1.
